# The use of laparoscopy in managing penetrating thoracoabdominal injuries in Africa: 83 cases reviewed

**DOI:** 10.1186/s13017-017-0137-2

**Published:** 2017-06-14

**Authors:** Zach M. Koto, Fusi Mosai, Oleh Y. Matsevych

**Affiliations:** Department of Surgery, Sefako Makgatho Health Sciences University (SMU), Pretoria, South Africa

**Keywords:** Thoracoabdominal injuries, Penetrating, Laparoscopy, Treatment, Africa

## Abstract

**Background:**

The use of laparoscopy in managing haemodynamically stable patients with penetrating thoracoabdominal injuries in developed countries is wildly practiced, but in Africa, the use of laparoscopy is still in its infancy stage. We reviewed a single centre experience in using laparoscopy in Africa for management of patients with both isolated diaphragmatic injuries as well as diaphragmatic injuries associated with intra-abdominal injuries requiring intervention.

**Methods:**

A retrospective analysis of prospectively collected data of patients presenting with penetrating thoracoabdominal injuries was done. All patients offered laparoscopic exploration and repair from January 2012 to December 2015 at Dr. George Mukhari Academic Hospital were analysed. Means (±SD) were presented for continuous variables, and frequencies (%) were presented for categorical variables. All analyses were performed using SAS version 9.3 (SAS Institute, Cary, NC).

**Results:**

A total of 83 stable patients with penetrating thoracoabdominal injuries managed with laparoscopy met the inclusion criteria and were included in the study. The Injury Severity Score ranged from 8 to 24, with a median of 18. The incidence of diaphragmatic injuries was 54%. Majority (46.8%) of patients had Grade 3 (2–10 cm defect) diaphragmatic injury. Associated intra-abdominal injuries requiring intervention were encountered in 28 (62%) patients. At least 93.3% of the patients were treated exclusively with laparoscopy. The morbidity was encountered in 7 (16%) patients; the most common cause was a clotted haemothorax Clavien-Dindo III-b, but only 1 patient required a decortication. There was one non-procedure-related mortality.

**Conclusions:**

A success rate of 93% in using laparoscopy exclusively was documented, with an overall 82% uneventful outcome. The positive outcomes found in this study when laparoscopy was used in stable patients with thoracoabdominal injuries support similar work done in other trauma centres. However, in addition, this study seem to suggest that the presence of peritonitis in stable patient is not a contra-indication to laparoscopy and thoracoscopy may be useful especially in right side diaphragmatic injury where the liver can preclude adequate visualization of the entire diaphragm and to thoroughly clean the chest cavity and prevent future complication such as residual clotted haemothorax.

*Clinical relevance*: The presence of peritonitis in stable patients with penetrating thoracoabdominal injury is not a contra-indication to laparoscopy provided the operating surgeon has adequate laparoscopic skills.

## Background

Management of patients with thoracoabdominal injuries forms a crucial part of our day to day management of trauma patients for a number of reasons. Missed traumatic diaphragmatic injuries (TDI) following penetrating thoracoabdominal injuries can result in catastrophic complications both in acute and chronic setting. These complications can range from asymptomatic diaphragmatic hernia to strangulated diaphragmatic hernia with associated high mortality rate of up to 8.8% [1].

The incidence of occult diaphragmatic injuries in asymptomatic patients with penetrating thoracoabdominal injuries is as high as 43% [2]. Even with the best current available imaging technology, a missed rate of occult TDI is as high as 50% [3]. In order to avoid missing these injuries, historically, these patients would be managed with mandatory exploratory laparotomy. But this approach resulted in non-therapeutic laparotomy rate as high as 33% [4]. The morbidity and mortality associated with non-therapeutic laparotomies is too high to justify this approach [5]. However, in the era of minimal access surgery, there is no justification for non-therapeutic laparotomies.

In the recent literature, the role of diagnostic laparoscopy has been demonstrated to be efficient and effective in assessing asymptomatic patients with penetrating thoracoabdominal injuries [6]. This approach has resulted in avoidance of non-therapeutic laparotomies [7, 8] However, some investigators would convert to open surgery once laparoscopy confirms peritoneal violation [4, 9]. But more recently, Mjoli et al. suggested that there is a therapeutic role of laparoscopy in patients with diaphragmatic injuries [10]. But in their study, the therapeutic intervention was only demonstrated in patients with no suspected associated intra-abdominal injuries (no peritonitis, no evisceration, and no free air) and only in left sided diaphragmatic injuries [10]

Rivaben et al. reported in his experimental study in animals an incidence of diaphragmatic hernia as high as 39% in right sided diaphragmatic injury [11]. Various contents were found in the hernia sac including small bowel, colon and stomach [11]. From this finding, we consider management of right sided thoracoabdominal injuries equally important.

The role of laparoscopy as an all-encompassing treatment strategy in managing all haemodynamically stable patients with penetrating thoracoabdominal injuries has not been well established. This treatment strategy includes stable patients with peritonitis, evisceration and free intra-abdominal air on pre-operative assessment.

In this study, we looked at the feasibility and safety of using laparoscopy in the treatment of haemodynamically stable patients with penetrating thoracoabdominal injuries in the following settings:

1. Isolated diaphragmatic injuries both left and right sided injuries.
2. Diaphragmatic injuries with associated intra-abdominal injuries requiring intervention including holow viscus perforation such as small bowel, colon and stomach with peritonitis.

## Methods

This study is a retrospective analysis of a prospectively collected data of patients presenting with penetrating thoracoabdominal injuries and were managed with laparoscopy in a trauma unit at Dr George Mukhari academic hospital (DGMAH). DGMAH is a tertiary hospital North-West of Pretoria, South Africa. All patients who were managed with laparoscopic exploration and/or repair from January 2012 to December 2015 were reviewed. Ethics clearance was obtained from Sefako Makgatho Health Sciences University (SMU) Research Ethics Committee (SMUREC) in accordance with Helsinki declaration.

All patients were initially managed according to the Advanced Trauma Life Support (ATLS) principles.

### Inclusion criteria

- Stable patients with penetrating thoracoabdominal injuries who were managed with laparoscopy
- With or without peritonitis
- Both left and right side penetrating thoracoabdominal injuries
- 12 years and above

### Exclusion criteria

- Penetrating thoracoabdominal injuries managed with laparotomy
- Blunt thoracoabdominal injuries
- Pregnancy
- Associated head injury

### Data collected

Demographic profile of the study population such as age and gender were documented. The mechanism of injury, number and the site of penetrating wounds, severity of the injury, cavity used to access the injury, intra-operative findings and grading, intra-operative complications and outcomes were documented.

Mechanism of injury was classified as either stab wound or gunshot wound. Numbers of penetrating wounds counted were only in the thoracoabdominal region, and the site was defined as left or right. Thoracoabdominal region was defined as the body region between upper border: from the 4th intercostal space in the mid-clavicular line anteriorly, the 6th intercostal space laterally in the mid-axillary line and the 8th intercostal space along the mid-scapular line and lower border: sub-costal margin, with the sternum and vertebral body forming the anterior and posterior medial borders. The severity of the injury was calculated using the Injury Severity Score (ISS). Cavity used either for diagnosis or intervention was documented as thoracoscopic, laparoscopic or both. Intra-operative findings were documented as follows: no injuries found, isolated diaphragmatic injury or diaphragmatic injury with associated injuries requiring intervention, the type of injury and the grading of the injury. Type of intervention was classified as exclusively laparoscopic repair or laparoscopic-assisted repair (hybrid procedure). Intra-operative complications were divided into procedure related or non-procedure related. Procedure-related complications are defined as complication caused directly by the procedure/surgeon such as iatrogenic bowel injuries.

Outcome variables measured were morbidity and mortality based of complications. Clavien-Dindo (CD) classification of surgical complication was used Appendix. The complications were sub-classified into procedure related and non-procedure related. Procedure-related morbidity and mortality were defined as those complications caused by the procedure/surgeon such as residual-clotted haemothorax requiring re-intervention, port-site hernia and anastomotic leaks.

Patients who did not have any documented morbidity or mortality were classified as uneventful outcomes.

### Operative procedure

All patients with thoracoabdominal injuries who were hemodynamically stable were offered laparoscopy under general anaesthesia. The thoracoabdominal injuries were defined as injuries that involved the body region between the nipple line or 4th intercostal space and the costal margin. The camera port was placed at the umbilicus, and the working ports were placed in the mid-clavicular line on both sides at level of the umbilicus. The entire abdominal cavity was inspected and checked for injuries, and where injuries are found, they were repaired laparoscopically. Post-operative care was done in the ward or high dependency area. Oral diet was commenced once the patient can tolerate the intake. The patient was discharged home once they can tolerate ward diet.

### Statistical analysis

Means (±SD) were presented for continuous variables, and frequencies (%) were presented for categorical variables. All analyses were performed using SAS version 9.3 (SAS Institute, Cary, NC).

## Results

**Fig. 1:**
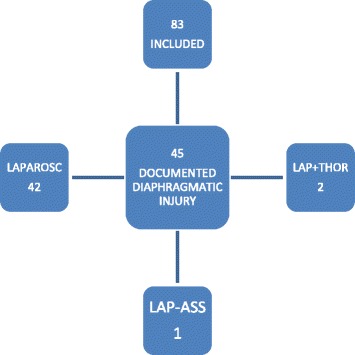
Study population

A total of 83 stable patients with penetrating thoracoabdominal injuries managed with laparoscopy met the inclusion criteria and were included in the study (Fig. 1). The median age was 26 years, with males accounting for 87% of the study population (Table 1). There were two mechanisms of injury noted, stab and gun shot. Majority (71%) were victims of stabs (Table 2).

**Table 1:** Patient’s characteristics

Gender	Frequency	Percentage	
F	6	13.3	
M	39	86.7	
Age	Minimum	Median	Maximum
	19	26	53

**Table 2:** Results

MOI	Stab	Gunshot		
	32 (71%)	13 (29%)		
Number of wounds	Single	Multiple		
	13 (29%)	32 (71%)		
Location of injury	Lower chest	Upper chest		
	44 (98%)	1 (2%)		
Site of injury	Left	Right	Left and right	
	34 (75.6%)	9 (20%)	2 (4.4%)	
ISS	Minimum	Median	Maximum	
	8	18	24	
Mode of intervention	Laparoscopy	Laparoscopic assisted	Laparoscopy-thoracoscopy	
	42 (93.3%)	1 (2.2%)	2 (4.4%)	
TDI grade	Grade 1 (contusion)	Grade 2 (≤2 cm)	Grade 3 (2–10 cm)	Grade 4 (>10 cm)
	3 (6.7%)	20 (44.5%)	21 (46.75)	1 (2.2%)
ASS injury (28 = 62%)	Stomach	Liver	Colon	Spleen
	13	11	5	3
Morbidity	Clotted haemothorax	Bleed	Abscess	Anastomotic leak
	5 (11%)	2 (4.4%)	1 (2.2%)	1 (2.2%)
Mortality	1 (2.2%)			

*MOI* mechanism of injury, *ISS* Injury Severity Score, *TDI* traumatic diaphragmatic injury, *ASS injury* Associated injury

The incidence of diaphragmatic injuries was 54% (Table 2). Majority (46.8%) of patients had Grade 3 (2–10 cm defect) diaphragmatic injury (Table 3), with 93.3% of the patients being treated exclusively with laparoscopy, 1 (2.2%) patient treated using laparoscopic-assisted approach (LAA) and 2 (4.4%) patients treated using both laparoscopy and thoracoscopy.

**Table 3:** Grading of diaphragmatic injury

Diaphragmatic injury	Frequency	Percent
Gr 1 (contusion)	3	6.67
Gr 2 (≤2 cm)	20	44.45
Gr 3 (2–10 cm)	21	46.67
Gr 4 (>10 cm)	1	2.22
Total	45	100.00

More than 70% of the patients sustained multiple injuries, with 20% of the site of the injury being the left sided diaphragmatic injuries. The Injury Severity Score ranges from 8 to 24, with a median of 18. Associated intra-abdominal injuries requiring intervention were encountered in 28 (62%) patients. The most common associated injury was the stomach, followed by the liver (Table 2). Six (21%) patients had more than one associated intra-abdominal injuries (Table 4). There were two intra-operative-related complications encountered, and both were due to bleeding. The most common post-operative complication was a clotted haemothorax CD III-b, and only 1 patient required a decortication (Table 5). There was one anaesthetic-related mortality.

**Table 4:** Associated injury and grading

ASS INJURY	Frequency	Percent	Cum. Percent
Gr 1 liver	3	6.67	6.67
Gr 1 spleen	2	4.44	11.11
Gr 2 colon	2	4.44	15.56
Gr 2 colon	1	2.22	17.78
Gr 2 colon, Gr 4 spleen	1	2.22	20.00
Gr 2 liver	1	2.22	22.22
Gr 2 liver	2	4.44	26.67
Gr 2 stomach	7	15.56	42.22
Gr 2 stomach, Gr 2 liver	1	2.22	44.44
Gr 2 stomach, Gr 2 spleen	1	2.22	46.67
Gr 2 stomach, Gr 3 colon	1	2.22	48.89
Gr 2 stomach, Gr 3 liver	1	2.22	51.11
Gr 3 colon	1	2.22	53.33
Gr 3 colon, Gr 4 liver	1	2.22	55.56
Gr 3 liver	1	2.22	57.78
Gr 3 stomach	1	2.22	60.00
Gr 3 stomach, Gr 2 liver	1	2.22	62.22
No associated injury	17	37.78	100.00
Total	45	100.00	100.00

**Table 5:** Clavien-Dindo grading

Grades	Definition	Management
I	Abscess—port-side	Opened at bedside
III-b	Anastomotic leak	Laparotomy and diverting stoma
III-b	Clotted haemothorax × 4	VATS × 4
V	Clotted haemothorax	Thoracotomy and decortication, died from anaesthetic complications

## Discussion

The incidence of diaphragmatic injuries was 54%; this is higher than the 7–48% reported in literature [10, 12–15]. The possible reason for this high incidence in our study is partly because we included stable patients with peritonitis and right side diaphragmatic injuries. All 46% of the patients who had negative laparoscopy were discharge 24 h after surgery, and there were no complications recorded at 1 and 3 weeks follow-up.

The wide spectrums of patients were represented as evidenced by wide range of ISS from 8 to 24. ISS has been identified as independent factor affecting the outcomes [16], high ISS has been wildly acknowledged as a predictor of mortality [17]. Majority of these patients had Grade 3 (2–10 cm defect) diaphragmatic injury with 62% of the patients having associated intra-abdominal injuries requiring intervention. Some authors report a lower incidence of about 50% [18]; in their methodology, most of these studies derived their figures from both blunt and penetrating injuries [19]. The incidence in our study is high because we only focused on penetrating thoracoabdominal injuries, which is associated with a higher incidence of diaphragmatic injuries than blunt thoracoabdominal injury [20].

Our incidence of associated intra-abdominal injuries requiring intervention was 62%; this includes both solid and hollow viscous organs. This figure is higher than 53.8% reported in other studies [14]. Associated injuries (abdominal and thoracic) are reported as a significant factor contributing to mortality in these patients [20]. Despite the high ISS and associated intra-abdominal injuries including the presence of peritonitis, 93% of patients were successfully treated using laparoscopy. This suggests that high ISS and having intra-abdominal-associated injuries are not a contra-indication to laparoscopy. It also demonstrates that with appropriate laparoscopic skill, these patients can still benefit from minimal access surgery.

Only 1 patient had multiple complex colonic injuries which required a laparoscopic-assisted approach. We still prefer a hybrid procedure if there are no compelling reasons to convert the patient to laparotomy. This particular patient had multiple hollow viscus injuries which included stomach and two colonic perforations in different areas, both requiring resection and anastomosis. The reason for laparoscopic-assisted approach in this particular case was to shorten the operating time.

Intra-operative complications were encountered in 2 patients (2.2%); both were due to iatrogenic bleeding. The bleeding was controlled laparoscopically. We considered this as a complication because both patients required blood transfusion post operatively. However, this operative morbidity is not higher than 2.4% reported by other authors [21].

Post-operative complication rate was 16%. The clotted haemothorax was responsible for 5 cases (11%) of post-operative complications, which is more than half of all post-operative complications. This was despite the insertion of an under water drainage. The common factor in all these patients was associated with lung injury. The explanation for these findings could either be residual clots left behind at the index operation or patients continued to ooze from associated lung injury post operatively or both factors played a role. However, we consider these complications as purely avoidable, and since these findings were made, we have been extra-cautious and we wash the thoracic cavity thoroughly during the index operation. However, all but 1 patient were successfully managed with suction and under water drainage. One patient had colonic anastomotic leak that was managed by bringing out a colonic stoma. Adhesive small bowel obstruction was seen after 5 months of discharge from the hospital in 1 patient. One patient developed port-site sepsis (abscess), which was managed with local dressings and wound care. Overall, this post-operative morbidity was not higher than 48% reported in other studies [22].

Thoracoscopy is not done routinely; however, in 2 patients from the study, it was deemed necessary because in 1 case, the patient had right side diaphragmatic injury with constant oozing of blood from the chest without a clear identifiable source. Thoracoscopy revealed injury in the bare area of the liver oozing into the chest cavity. Both the liver and the diaphragm were sutured using thoracoscopic approach. The second case of thoracoscopy was done because the right side-clotted haemothorax could not be evacuated properly using laparoscopy approach due to the liver obstructing the view.

There was one (2.2%) mortality; this patient had multiple injuries in the colon as well as lung injury. The patient recovered from the index operation and was discharged from the hospital. About 2 months later, the patient presented with clotted haemothorax, which required thoracotomy for decortication. Unfortunately, the patient died from anaesthetic complications following a thoracotomy. Combined abdominal and thoracic injury in patients with TDI is notoriously associated with high mortality [20]. However, our mortality was still lower than 23% which was reported in literature [18].

Overall, 82% of the patients had uneventful outcome and there were no missed hollow viscus injuries.

### The rationale for excluding patients with associated closed head injury from the study

Currently, there is no published literature as far as we are aware which demonstrates the safety of laparoscopy in patients with closed head injury. Authors have documented changes/worsening of ICP due to pneumoperitonium when laparoscopy is used in large animal models, and they raised serious concerns about the use of laparoscopy in these patients [23, 24]. Even though there are no prospective human trials on this issue, Mobbs and Ow Yang published a case report where ICP in closed head injury patient worsened from 9 to 60 mmHg within 10 min of pneumoperitonium [25] and Kamine et al. also raised a concern after retrospective analysis of patients who underwent VP-shunt and had abdominal insufflation with CO_2_ [26, 27]. Therefore, due to uncertainty regarding the safety of laparoscopy in head injury patients, we opted to exclude these patients from the study for safety reasons. However, we do concede that further studies need to be done on this topic.

## Conclusion

The positive outcomes found in this study demonstrate the feasibility of laparoscopy when used in stable patients with penetrating thoracoabdominal injuries. However, in addition, this study seems to suggest that the presence of peritonitis in a stable patient is not a contra-indication to laparoscopy. Thoracoscopy may be useful especially in right side diaphragmatic injury where the liver can precludes adequate visualization of the entire diaphragm and to thoroughly clean the chest cavity and prevent future complication.

## Appendix

### Clavien-Dindo Classification

‡ brain hemorrhage, ischemic stroke, subarachnoid bleeding, but excluding transient ischemic attacks (TIA); IC: Intermediate care; ICU: Intensive care unit.

Dindo D, Demartines N, Clavien PA. Classification of surgical complications: a new proposal with evaluation in a cohort of 6336 patients and results of a survey. Ann Surg. 2004;240(2):205–13.
